# Efficacy of Powered Intracapsular Tonsillectomy: A Single-Blinded Prospective Study

**DOI:** 10.7759/cureus.93106

**Published:** 2025-09-24

**Authors:** Yoshiyuki Sasano, Kenichiro Tanabe, Nayu Yokoyama, Yuki Kitajima, Taro Inagaki, Fumihiro Mochizuki, Manabu Komori

**Affiliations:** 1 Otolaryngology, St. Marianna University School of Medicine, Kawasaki, JPN; 2 Graduate School of Medicine, St. Marianna University School of Medicine, Kawasaki, JPN

**Keywords:** obstructive sleep apnea (osa), powered intracapsular tonsillectomy, recurrent tonsillitis, tonsillectomy, tonsillectomy outcomes

## Abstract

Background

Powered intracapsular tonsillectomy (PIT) is considered a less invasive alternative to conventional extracapsular tonsillectomy (cTE), potentially reducing postoperative pain and complications. However, most prior studies have been retrospective.

Objective

This study aimed to assess the clinical utility of PIT in comparison to cTE through a single-blind, randomized controlled trial.

Methods

In this prospective trial (registered with the WHO International Clinical Trials Registry Platform, ID: JPRN-UMIN000043776), patients undergoing PIT (n = 6) or cTE (n = 5) were evaluated. The primary outcome was postoperative pain measured by the FACES Pain Rating Scale (FRS). Secondary outcomes included analgesic use, dietary intake, and postoperative bleeding.

Results

The PIT group showed a trend toward lower FRS scores on postoperative days two to four (p = 0.053) and significantly reduced analgesic consumption (p < 0.001). No significant differences were found in dietary intake. No postoperative bleeding occurred in either group.

Conclusion

PIT was associated with significantly lower analgesic use and a trend toward reduced postoperative pain, with no increased risk of bleeding. PIT may be a safe and minimally invasive alternative to cTE, potentially facilitating earlier postoperative dietary advancement.

## Introduction

Tonsillectomy is a widely performed procedure across a broad age range, from children to adults, for various indications, including obstructive sleep apnea (OSA), recurrent tonsillitis, and focal infections such as IgA nephropathy. Among otolaryngological surgeries, tonsillectomy is one of the most frequently conducted. However, conventional extracapsular tonsillectomy (cTE) is associated with considerable postoperative pain, often resulting in difficulty with dietary intake and necessitating the use of analgesics, which are sometimes insufficiently effective. Poor dietary intake may further contribute to malnutrition and delayed wound healing.

Postoperative hemorrhage, a common complication, occurs in approximately 6.3% to 14% of cases [1,2], depending on institutional definitions and criteria. When it occurs, it may require readmission or surgical intervention for hemostasis, and in rare cases, it can be life-threatening. In pediatric patients, the risks of postoperative airway obstruction, dehydration, and secondary hemorrhage are especially pronounced [3]. Thus, a safer alternative to cTE is desirable, particularly in children.

Recently, powered intracapsular tonsillectomy (PIT), using devices such as coblators and microdebriders, has gained international adoption. Several retrospective studies have suggested that PIT may result in less postoperative pain and a lower incidence of bleeding compared to cTE [4]. However, randomized controlled trials are limited. This study aimed to evaluate the postoperative safety of PIT compared to cTE in terms of pain and hemorrhage using a single-blinded randomized controlled design.

## Materials and methods

Subjects and outcome measures

This single-blinded randomized controlled trial included 11 patients aged five to 60 years who underwent tonsillectomy at our institution for OSA or recurrent tonsillitis between February 2023 and November 2024. Exclusion criteria included IgA nephropathy, periodic fever with aphthous pharyngitis, and adenitis; patients with these conditions, for whom complete tonsillectomy would be preferable, were therefore excluded. Participants with a history of peritonsillar abscess were included in the recurrent tonsillitis group. Written informed consent was obtained from all adult participants and from legal guardians for pediatric participants, with assent from children where appropriate. Patients aged five to 15 years were categorized as pediatric, and those aged 16-60 years as adults. Stratified permuted block randomization was used, stratified by underlying condition (OSA or recurrent tonsillitis), to assign participants to the PIT or cTE group. Patients were blinded to group assignment; participants aged 16 years and older were not informed of whether PIT or cTE had been performed until after study evaluation was completed. For participants aged 15 years and younger, only their legal guardians were informed of the procedure performed during the postoperative explanation. The observation period extended from the day of surgery (day one) to day 29 (±7). Patients were hospitalized through day eight and then followed on an outpatient basis on days 15 (±4) and 29 (±7).

The primary outcome was the mean FACES Pain Rating Scale (FRS) score from days two to four. Permission to use the FRS for this study was obtained by the authors. Secondary outcomes included total analgesic use from days two to four, average dietary intake from days two to four, and incidence of postoperative hemorrhage throughout the study period.

Postoperative pain was assessed once daily in the morning using the six-point FRS (zero to five) [5]. Analgesia consisted of acetaminophen 500 mg or loxoprofen sodium 60 mg as needed for adults and acetaminophen granules (20%) at 10 mg/kg for pediatric patients. Analgesics were permitted every four hours, as needed, up to four times daily. Intravenous acetaminophen (1000 mg for adults, 10 mg/kg for children) was administered at the end of anesthesia.

Meals were served three times daily, each comprising a main and a side dish. Postoperative dietary management followed our institutional protocol: patients were kept fasting on day zero, started on a liquid diet from postoperative day one, and from day one to day five, the proportion of gruel was gradually increased. A full gruel diet was provided on day five, and a regular-texture diet was resumed on day seven. Patients were also instructed to avoid hard foods and sticky foods, as well as oily meals, until day 15 (±4). Dietary intake was scored separately on an 11-point scale (0:0%, 1:10%-10:100%), and the total was averaged by the number of meals recorded for each day.

Postoperative hemorrhage was defined as visible clotting or active bleeding on examination, or patient-reported bleeding episodes, and classified by Windfuhr’s grading system (Grades 1-5) [1].

This study was approved by the Ethics Committee of St. Marianna University School of Medicine (Approval No. 5641) and administered by the WHO International Clinical Trials Registry Platform (ID: JPRN-UMIN000043776).

Surgical techniques

The surgeries were performed by three otolaryngologists, each with more than six years of clinical experience.

Conventional Extracapsular Tonsillectomy (cTE)

Under general anesthesia, a mouth gag was used to secure the surgical field. The tonsil was grasped with a Heymann septum forceps, and resection was performed under direct vision using a combination of cold instruments (e.g., scalpel, dissector, cotton balls) and hot instruments like a bipolar electrocautery following the hot-and-cold tonsillectomy technique. Hemostasis was achieved with bipolar cautery as needed.

Powered Intracapsular Tonsillectomy (PIT)

Under general anesthesia, the surgical field was visualized using a mouth gag and a rigid endoscope (0° or 30°). A 40° Radenoid blade (Medtronic, Dublin, Ireland), rotating at 600-800 rpm, was used to excise the tonsil. When the tonsillar tissue, particularly the upper pole, was deeply embedded, the tonsillar tissue was pulled out with a Heymann septum forceps, and resection proceeded. Approximately 80-90% of the tonsillar tissue was resected. Bleeding sites were initially compressed gently with cotton; if hemostasis was insufficient, bipolar electrocautery was used sparingly.

Statistical analysis

Pain Score

The primary analysis compared treatment groups using a general linear model with mean pain score as the objective variable and underlying disease as the covariate variable. In addition, an exploratory analysis was performed using a mixed effects model for repeated measures (MMRM) with pain score on each postoperative day as the objective variable, treatment group, postoperative day, underlying disease, and interaction of treatment group and postoperative day as fixed effects, and patient as a random effect.

Analgesic Use

Since the total number of analgesic doses is count data, treatment groups were compared using a Poisson regression model with the total number of analgesic doses as the objective variable, the underlying disease as the covariate variable, and the link function as the natural logarithm function.

Dietary Intake

Treatment groups were compared using a general linear model with the mean dietary intake as the objective variable and the underlying disease as the covariate variable. In addition, exploratory analyses were performed using an MMRM with dietary intake on each postoperative day as the objective variable, treatment group, postoperative day, underlying disease, and interaction of treatment group and postoperative day as fixed effects and patient as random effects.

All analyses were pre-specified in the statistical analysis plan. Hypothesis testing was two-tailed, with statistical significance set at p < 0.05. No statistical adjustment was made for multiplicity of tests. All statistical analyses were performed using SAS version 9.4 (SAS Institute Inc., Cary, NC, USA).

## Results

Patient characteristics

Eleven patients were enrolled: six in the PIT group (four pediatric, two adult) and five in the cTE group (four pediatric, one adult). None had a history of comorbidities. Patient characteristics are summarized in Table 1. 

**Table 1:** Background of patients. PIT: powered intracapsular tonsillectomy; cTE: conventional extracapsular tonsillectomy; OSA: obstructive sleep apnea

Parameters		PIT			cTE		
		Total	Male	Female	Total	Male	Female
N		6(100%)	3(50.0%)	3(50.0%)	5(100%)	2(40.0%)	3(60.0%)
Age		13.5 ±11.0	14.0±11.3	13.0±8.5	16.8 ±18.1	7.00±1.0	18.2±23.3
Age group	Adult (16–60 years)	2(33.3%)	1(33.3%)	1(33.3%)	1(20.0%)	0(0%)	1(33.3%)
	Child (5–15 years)	4(66.7%)	2(66.7%)	2(66.7%)	4(80.0%)	2(100%)	2(66.7%)
Present illness	OSA	4(66.7%)	2(66.7%)	2(66.7%)	4(80.0%)	2(100%)	2(66.7%)
	Recurrent tonsillitis	2(33.3%)	1(33.3%)	1(33.3%)	1(20.0%)	0(0%)	1(33.3%)

Pain scores

**Figure 1:**
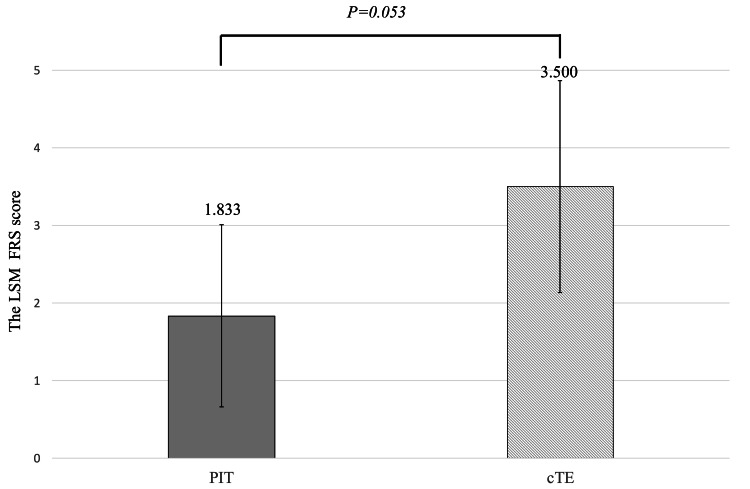
LSMs of the FRS on days two to four for the two groups. Treatment groups were compared using a general linear model with mean pain score as the objective variable and the underlying disease as the covariate variable, with p-values calculated using this model. FRS: FACES Pain Rating Scale; LSM: least squares mean; PIT: powered intracapsular tonsillectomy; cTE: conventional extracapsular tonsillectomy

Figure 1 shows the least squares mean (LSM) FRS scores on days two to four. Although the PIT group tended to report lower pain scores than the cTE group, the difference was not statistically significant (LSM difference: -1.667; p = 0.053). 

**Figure 2:**
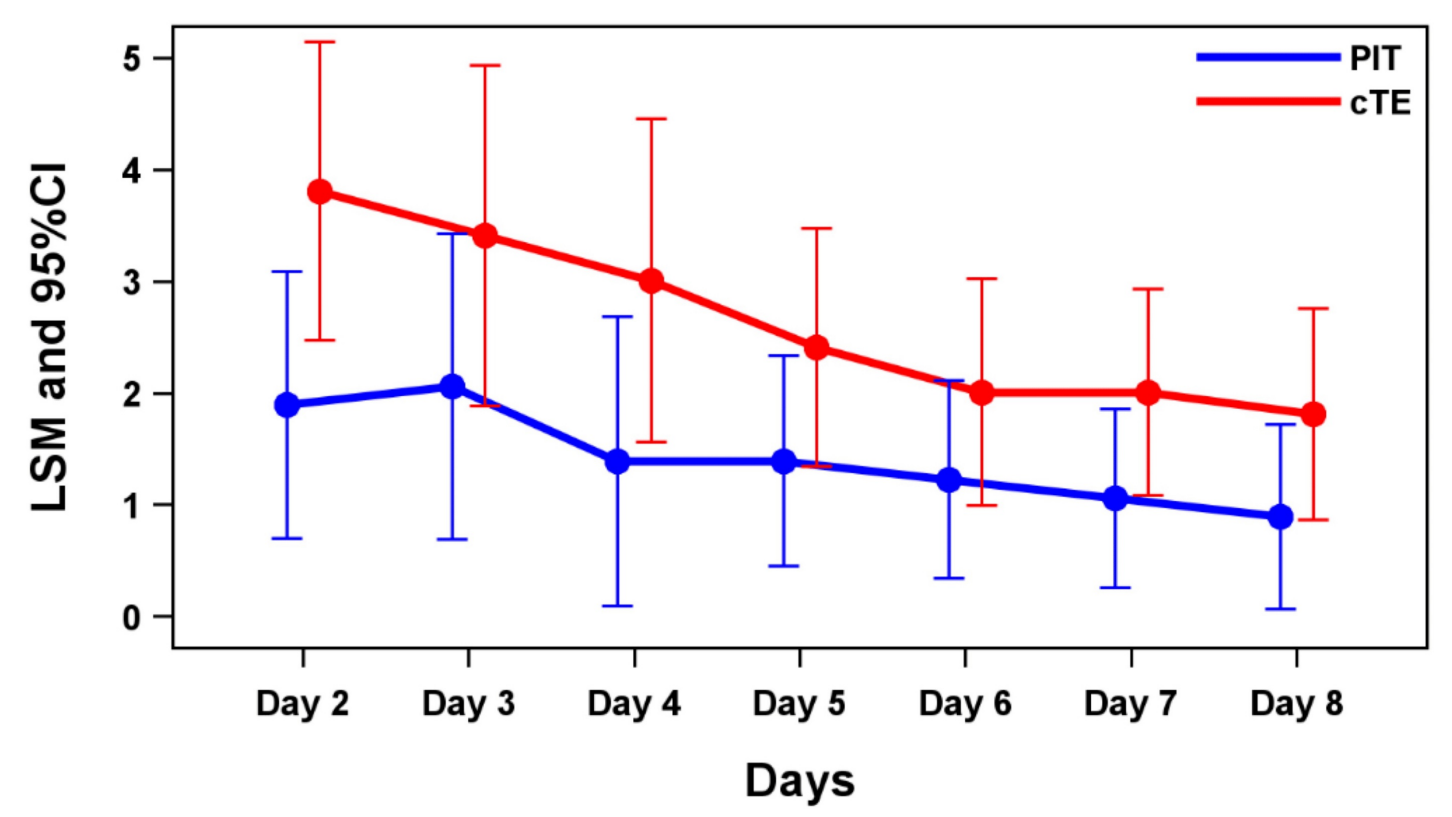
LSMs of the FRS and 95% CIs for MMRM on days two to eight. FRS: FACES Pain Rating Scale; LSM: least squares mean; MMRM: model for repeated measures; PIT: powered intracapsular tonsillectomy; cTE: conventional extracapsular tonsillectomy

Figure 2 and Table 2 present LSMs and 95% confidence intervals (CIs) for FRS scores from days two to eight based on the MMRM analysis. The MMRM LSMs of the FRS at day two were -1.915 lower in the PIT group than in the cTE group (p = 0.036).

**Table 2:** The number of subjects included for each day. PIT: powered intracapsular tonsillectomy; cTE: conventional extracapsular tonsillectomy

Group	Day2	Day3	Day4	Day5	Day6	Day7	Day8
PIT	6	6	6	6	6	6	6
cTE	5	5	5	5	5	5	5

Analgesic use

**Figure 3:**
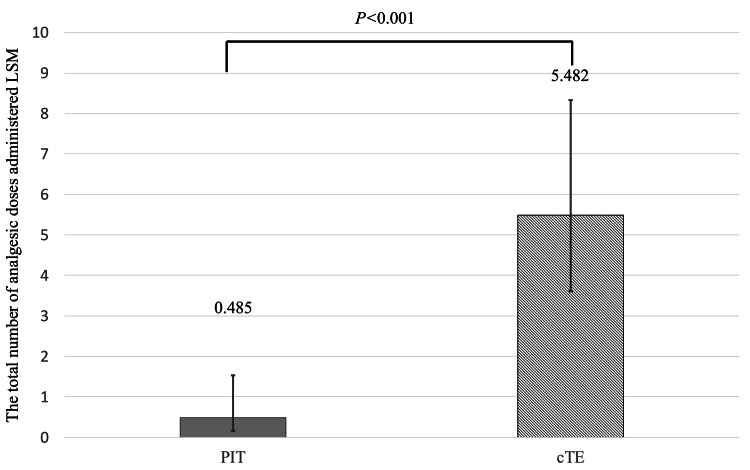
Total number of analgesic doses administered (LSM) on days two to four for the two groups. Treatment groups were compared using a Poisson regression model with the total number of analgesic doses as the objective variable, the underlying disease as the covariate variable, and the link function as the natural logarithm function, with p-values calculated using this model. LSM: least squares mean; PIT: powered intracapsular tonsillectomy; cTE: conventional extracapsular tonsillectomy

Figure 3 displays the total number of analgesic doses administered LSM between days two and four. The PIT group used significantly fewer analgesics compared to the cTE group (LSM ratio: 0.089; p < 0.001). 

Dietary intake

**Figure 4:**
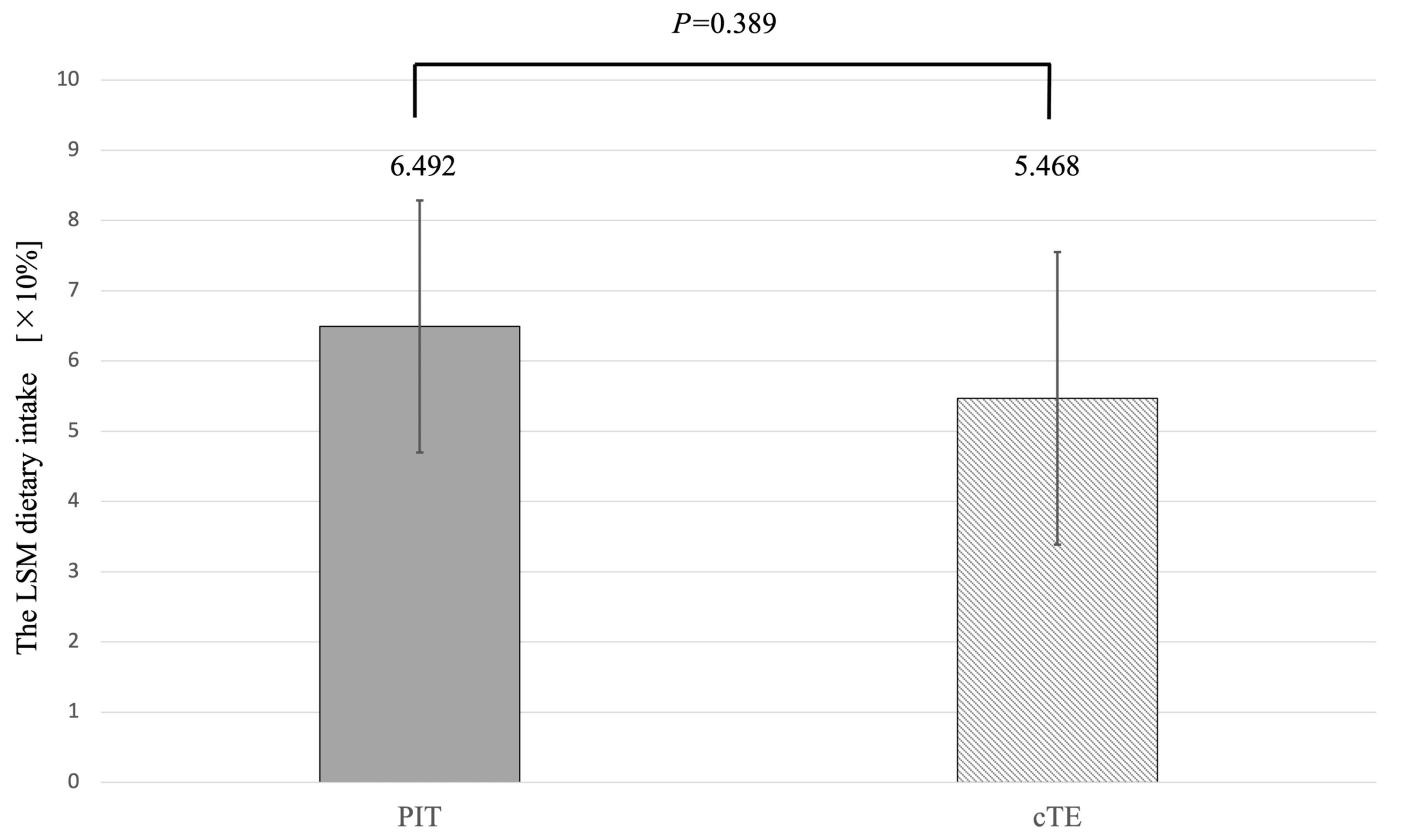
LSMs of the dietary intake on days two to four for the two groups. Dietary intake was scored in 10% increments (0:0%, 1:10% to 10:100%), and six daily measurements (three each of main and side dishes) were averaged for each day. Treatment groups were compared using a general linear model with the mean dietary intake as the objective variable and the underlying disease as the covariate variable, with p-values calculated using this model. LSM: least squares mean; PIT: powered intracapsular tonsillectomy; cTE: conventional extracapsular tonsillectomy

Figure 4 shows the LSM dietary intake from days two to four. No statistically significant difference was observed between groups (LSM difference: 1.024; p = 0.389). 

**Figure 5:**
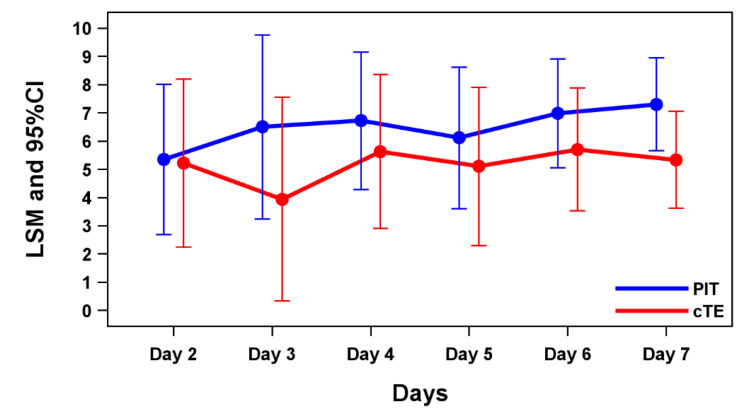
LSMs of dietary intake and 95% CIs for MMRM on days two to seven. LSM: least squares mean; MMRM: model for repeated measures; PIT: powered intracapsular tonsillectomy; cTE: conventional extracapsular tonsillectomy

Figure 5 and Table 3 present LSMs and 95% CIs for dietary intake from days two to seven using the MMRM model. No significant intergroup differences were found on any day. Although patients were hospitalized through day eight, day seven data are presented as all patients were discharged on the morning of day eight. 

**Table 3:** The number of subjects included for each day. On day seven, one subject in each of the PIT and cTE groups was excluded from the analysis due to missing data. PIT: powered intracapsular tonsillectomy; cTE: conventional extracapsular tonsillectomy

Group	Day2	Day3	Day4	Day5	Day6	Day7
PIT	6	6	6	6	6	4
cTE	5	5	5	5	5	4

Postoperative hemorrhage

No postoperative hemorrhage occurred within 24 hours or during the entire follow-up period in either group.

## Discussion

Vicini et al. retrospectively compared 251 patients who underwent PIT with 199 patients who underwent cTE, reporting a significantly lower average visual analog scale score over the first three postoperative days in the PIT group (4.1 vs. 6.3) [6]. In the present study, although FRS scores between days two and four did not show a statistically significant difference, exploratory analysis revealed that the MMRM LSM of pain score on day two was -1.915 lower in the PIT group than in the cTE group (p = 0.036), suggesting a trend toward reduced postoperative pain.

Regarding analgesic use, Bender et al. retrospectively analyzed 104 adults undergoing tonsillectomy for chronic tonsillitis and found significantly reduced analgesic requirements in the PIT group (p < 0.05) [7]. Similarly, Korkmaz et al. conducted a prospective randomized trial comparing 40 children with OSA undergoing partial intracapsular tonsillectomy to 41 receiving cTE, reporting significantly fewer analgesic administrations in the PIT group (1.18 ± 1.27 vs. 2.00 ± 1.45) [8]. Our findings are consistent with these reports. The reduced postoperative pain and analgesic use observed in the PIT group may be attributed to the avoidance of extracapsular dissection.

No significant difference in dietary intake was observed between the groups. This may reflect our institution's conservative postoperative nutritional protocol. Due to concerns about postoperative pain, our hospital has a policy of gradually changing the postoperative diet from a liquid diet to a full gruel diet on day five and a normal diet on day seven. In particular, younger children may refuse soft or porridge-like foods, potentially influencing intake scores. Given the reduced analgesic usage in the PIT group, earlier dietary advancement may be feasible in future practice. A systematic review by Zhang et al., encompassing 19 randomized controlled trials and 13 observational studies, reported that PIT shortened the time to resumption of a regular diet by 2.8 days compared to cTE [9], supporting the potential for accelerated postoperative recovery and reduced complications such as dehydration.

No cases of postoperative hemorrhage were observed in either group. Vicini et al. reported a hemorrhage rate of 6.8% in the cTE group and 0.7% in the PIT group in a retrospective study of 450 pediatric OSA patients [6]. Similarly, Noda et al. observed significantly lower bleeding rates in the PIT group (2.15%) compared to the cTE group (11.1%) in 174 children [10]. While our study did not demonstrate such differences, possibly due to the small sample size, the minimal use of hemostatic intervention in PIT did not increase bleeding.

This study has several limitations. First, the present study was conducted on a small number of cases, which limits the ability to detect significant differences. One reason is that tonsillectomy for OSA has become younger in recent years, with an increasing number of procedures performed at age three years or younger [11]. The FRS in this study was designed for children three years and older. Still, there is a report that it is difficult to reliably evaluate self-reported pain intensity and scale in patients under three years of age [12]. Therefore, in this study, which focused on postoperative pain as the primary outcome, we set the inclusion criterion at age five years or older. However, as our institution is a university hospital that treats relatively young patients, the predominance of patients under four years old was a major reason for the limited sample size in this study. In addition, the present study also included adult cases with recurrent tonsillitis as the underlying condition. In such patients, a “scarred tonsil” state often leads to firm adhesion, which may influence capsule preservation in PIT and cause additional damage to the muscular layer in cTE. Given the small sample size, these factors may have affected postoperative outcomes. Importantly, although the sample size was small, this study highlights valuable findings regarding postoperative pain, reduced analgesic requirements, and the potential for earlier dietary advancement.

Second, surgical visualization differed between groups: cTE was performed without surgical loupes or a microscope, whereas PIT was performed with endoscopic assistance. The magnified surgical field and clearer visualization might have contributed not only to capsule preservation but also to reduced damage to the muscular layer, possibly influencing the results.

Finally, the follow-up period was only one month. Tonsillar regrowth is a known long-term risk of PIT. Doshi et al. reported tonsillar regrowth in 33 of 559 children (5.9%) who underwent PIT with a microdebrider, with five requiring reoperation [13]. In contrast, Soaper et al. retrospectively reviewed 1456 PIT and 1052 cTE cases over an average of 8.2 years (maximum 14 years), reporting regrowth requiring surgery in only three PIT cases (0.21%), with no significant difference between groups (p = 0.4073) [14]. Nonetheless, further long-term studies are needed to fully evaluate the risk of regrowth after PIT.

## Conclusions

PIT was associated with a reduced frequency of postoperative analgesic use and showed a trend toward decreased postoperative pain compared to conventional tonsillectomy. These findings suggest that earlier advancement to a regular diet may be feasible following PIT. However, given the limited sample size, the present study should be regarded as exploratory, underscoring the necessity for additional case accumulation, multicenter investigations, and extended follow-up. Nevertheless, PIT appears to represent a safe and minimally invasive alternative to cTE.
